# Mammographic density and structural features can individually and jointly contribute to breast cancer risk assessment in mammography screening: a case–control study

**DOI:** 10.1186/s12885-016-2450-7

**Published:** 2016-07-07

**Authors:** Rikke Rass Winkel, My von Euler-Chelpin, Mads Nielsen, Kersten Petersen, Martin Lillholm, Michael Bachmann Nielsen, Elsebeth Lynge, Wei Yao Uldall, Ilse Vejborg

**Affiliations:** Department of Radiology, Copenhagen University Hospital, Rigshospitalet, Blegdamsvej 9, DK-2100 Copenhagen Ø, Denmark; Department of Public Health, University of Copenhagen, Øster Farimagsgade 5, DK-1014 Copenhagen K, Denmark; Department of Computer Sciences, University of Copenhagen, Universitetsparken 5, DK-2100 Copenhagen Ø, Denmark; Biomediq, Fruebjergvej 3, DK-2100 Copenhagen Ø, Denmark

**Keywords:** Mammographic breast density, Mammographic parenchymal pattern, BI-RADS density, Tabár, Mammographic texture, Breast cancer, Risk prediction

## Abstract

**Background:**

Mammographic density is a well-established risk factor for breast cancer. We investigated the association between three different methods of measuring density or parenchymal pattern/texture on digitized film-based mammograms, and examined to what extent textural features independently and jointly with density can improve the ability to identify screening women at increased risk of breast cancer.

**Methods:**

The study included 121 cases and 259 age- and time matched controls based on a cohort of 14,736 women with negative screening mammograms from a population-based screening programme in Denmark in 2007 (followed until 31 December 2010). Mammograms were assessed using the Breast Imaging-Reporting and Data System (BI-RADS) density classification, Tabár’s classification on parenchymal patterns and a fully automated texture quantification technique. The individual and combined association with breast cancer was estimated using binary logistic regression to calculate Odds Ratios (ORs) and the area under the receiver operating characteristic (ROC) curves (AUCs).

**Results:**

Cases showed significantly higher BI-RADS and texture scores on average than controls (*p* < 0.001). All three methods were individually able to segregate women into different risk groups showing significant ORs for BI-RADS D3 and D4 (OR: 2.37; 1.32–4.25 and 3.93; 1.88–8.20), Tabár’s PIII and PIV (OR: 3.23; 1.20–8.75 and 4.40; 2.31–8.38), and the highest quartile of the texture score (3.04; 1.63–5.67). AUCs for BI-RADS, Tabár and the texture scores (continuous) were 0.63 (0.57–0–69), 0.65 (0.59–0–71) and 0.63 (0.57–0–69), respectively. Combining two or more methods increased model fit in all combinations, demonstrating the highest AUC of 0.69 (0.63-0.74) when all three methods were combined (a significant increase from standard BI-RADS alone).

**Conclusion:**

Our findings suggest that the (relative) amount of fibroglandular tissue (density) and mammographic structural features (texture/parenchymal pattern) jointly can improve risk segregation of screening women, using information already available from normal screening routine, in respect to future personalized screening strategies.

**Electronic supplementary material:**

The online version of this article (doi:10.1186/s12885-016-2450-7) contains supplementary material, which is available to authorized users.

## Background

Breast cancer remains the most common malignancy among women worldwide, and is still the leading cause of female cancer death in most European countries [1].

Mammography screening has proved to decrease breast cancer mortality [2, 3]. Accordingly, breast cancer mortality was reduced by 25 % in screening targeted women (37 % for women participating) in the first 10 years of the Copenhagen Screening Programme [4]. Yet, two-view mammography is not perfect due to limited sensitivity and specificity particularly in women with dense breast tissue [5–8]. Not only does increased breast density reduce mammographic sensitivity, but it has also been firmly established as a strong risk factor for breast cancer. It has been shown that women with high density (>75 %) have a 4–6 times increased risk of breast cancer compared with women with low density (<5 %) [7, 9]. Personalized screening strategies based on a woman’s risk and mammographic sensitivity profile—including mammographic density assessment—is much debated [10–13], and informing screening-attendees of their BI-RADS density has today been covered by legislation in more than 20 US states, intending to improve screening for high-density-women [14, 15].

Traditionally, mammographic density is measured semi-quantitatively using the BI-RADS density classification [16] or quantitatively as an area-based percentage of mammographic density with Cumulus-like techniques [17, 18]. However, numerous newer techniques are gaining ground including fully automated volumetric measures (e.g. Volpara and Quantra) [19–24] as well as methods for density assessment using other modalities such as digital breast tomosynthesis (DBT), MRI, photon counting spectral mammography or ultrasound [25–27]. Still, the BI-RADS density classification remains the only density method in common clinical use. Currently, it is not fully understood if the established association with breast cancer is contributed by both the (relative) amount—density—but also the mammographic structural appearance (texture/parenchymal pattern). The Wolfe and Tabár classifications [28, 29] are examples of more qualitative radiological methods. However, in recent years a range of new automated measures of mammographic risk capturing textural/structural aspects of mammographic density have been introduced [30–37], which besides being associated with risk may improve risk segregation using density parameters alone [30, 31, 34].

**Fig. 1:**
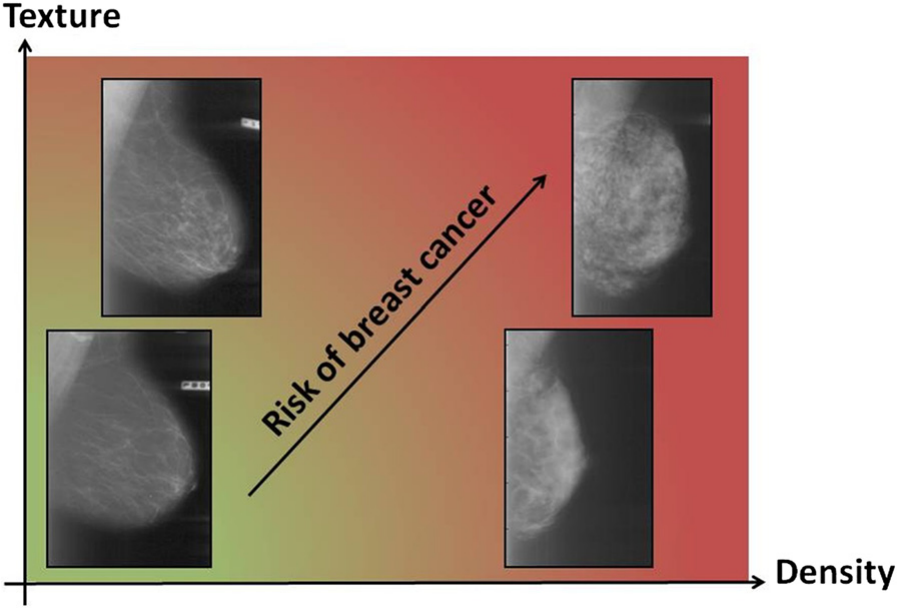
Density and texture as potential complementary mammographic risk markers. It may be hypothesized that measures of the (relative) amount of fibroglandular tissue and measures of the structural appearance of the fibroglandular tissue (density and texture) may both contribute to mammography detected risk. Increasing density and increasing texture may independently add to the risk of breast cancer (visualised as changes from the green colour zone to the light green/light red colour zone). Low density + low texture indicate the lowest mammographic risk (*green colour*) whereas high density + high texture indicate the highest risk (*red colour*). Combining these two risk markers could potentially improve risk segregation of screening women

The objectives of this study were 1) to relate three methods measuring density or corresponding structural appearance on digitized film-based mammograms using two well established radiological methods (the BI-RADS density classification—semi-quantitative 4th edition—and Tabar’s classification on parenchymal patterns) and a new fully automated texture quantification technique (in this paper referred to as *M*ammographic *T*exture *R*esemblance, MTR), and 2) to investigate to what extent quantification of mammographic structural appearance independently and jointly with density can improve prediction of future breast cancer in screening women, Fig. 1. We hypothesized that all three methods can individually segregate women into different risk groups, and that density and texture measurements on negative screening mammograms can jointly improve risk segregation.

## Methods

### Study population and mammograms

**Fig. 2:**
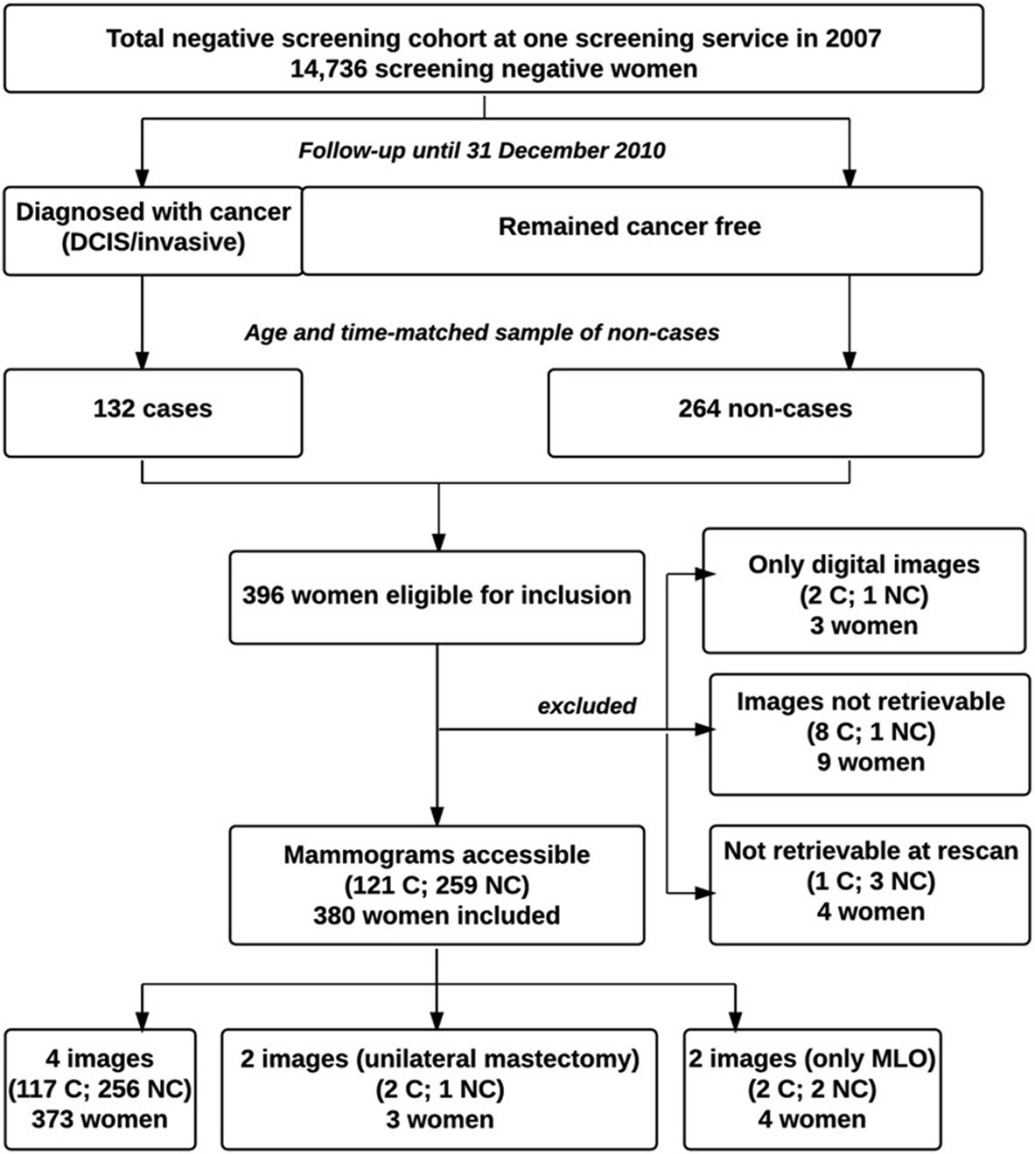
Flowchart of study design and population

The design and population of this nested case–control study, summarised in Fig. 2, have been described in detail previously [38]. In brief, our study cohort consisted of all 14,736 women with a negative screening mammogram (no cancer detected) in 2007—the last year with analogue mammography—attending biennial routine breast screening in a population-based screening programme in Copenhagen, Denmark. The women were followed until 31 December 2010. Information on death, emigration and/or histologically verified breast cancer or ductal carcinoma in situ (DCIS) were retrieved and linked from the following registers: the Danish Civil Registration System (CRS), the Danish Cancer Registry, the Pathology Registry and the Danish Breast Cancer Cooperative Group (DBCG). In total, 132 women were diagnosed with invasive breast cancer or DCIS. For each case, two controls matched on year of birth were selected from the cohort based on incidence density sampling [39]. Mammograms were not accessible for 16 women leaving 380 women for the final analyses.

Use of screening data and tumour-related information was approved by the Danish Data Inspection Agency (2013–41–1604). This is an entirely register based study and hence neither written consent nor approval from an ethics committee was required under Danish Law.

The craniocaudal (CC) and mediolateral oblique (MLO) projections from each breast were digitized using a Vidar Diagnostic PRO Advantage scanner (Vidar systems corporation, Herdon, VA, USA) providing an 8-bit (256 grey scales) output at a resolution of 75–150 DPI. These images were assessed radiologically. However, a higher resolution is required for fully automated computerized techniques. Thus, to assess the automated MTR scores, mammograms were re-scanned on an equivalent Vidar Diagnostic PRO Advantage scanner providing a 12-bit (4096 grey scales) output at a resolution of 570 DPI with upgraded software (eFilm Scan 2.0.1 Build 586). At rescanning images from four women could not be recovered and were excluded from the present study (Fig. 2).

### Mammographic classification

The digitized mammograms were classified according to two radiological methods: The *4th edition* of the American College of Radiology (ACR)’s Breast Imaging-Reporting and Data System (BI-RADS) density classification [40] and the Tabár classification on parenchymal patterns [29, 41]. Both classification schemes were detailed in Winkel et al. (2015) [38]. In brief, the BI-RADS density classification assigns mammograms semi-quantitatively into four categories: ***D1***: fatty *(<25 % fibro-glandular tissue)*, ***D2***: scattered fibro-glandular densities *(25–50 %)*, ***D3***: heterogeneously dense *(51–75 %)* and ***D4***: extremely dense *(>75 %)* [40]. The Tabár classification is based on a histological-mammographic correlation and mammograms are assigned into five more descriptive/qualitative categories: ***PI***: Scalloped contours with oval-shaped lucencies and evenly scattered 1–2 mm nodular densities, ***PII***: Almost complete fatty replacement, ***PIII***: Like *PII* with a retroareolar prominent duct pattern (representing periductal connective tissue proliferation or distended fluid-filled ducts), ***PIV***: Prominent nodular and linear densities with nodular densities larger than normal lobules (representing a variety of changes i.e. adenosis or fibrosis) and ***PV***: Dominated by homogeneous, ground glass like and nearly structure-less densities (representing extensive fibrosis) [29, 41]. Two MDs—a senior breast radiologist (5 years full-time experience in breast radiology) and a resident in radiology (no previous experience in breast radiology)—independently classified the randomized mammograms according to the two radiological methods. More precise density measures are achieved when mentally fusing two projections compared with assessing only a single projection of the breast. Therefore, CC and MLO views were evaluated together equal to clinical practise. Evaluation by the Tabár classification was done blinded from the BI-RADS assessment (separated in time) in order to reduce artificial agreement between the two methods. The readers were also blinded to the original mammographic reading, the date of examination, the woman’s age and case/control-status. Inter-observer reproducibility on the two manual methods (based on each breast) was *substantial* demonstrating kappa values of 0.68 (0.64–0.72) and 0.64 (0.60–0.69) for BI-RADS and Tabár, respectively [38]. For statistical analyses, consensus scores were obtained if the two readers disagreed.

Subsequently, all mammograms were assessed by a fully automated mammographic texture resemblance marker (denoted MTR) [42]. The MTR scores were calculated using a deep learning convolutional neural network pipeline by Biomediq [42]. Initially, a number of mammogram specific texture building blocks were trained in an unsupervised manor (using no cancer label information) from a large collection of mammograms. Then, we used patches from a database of diagnosis-free mammograms with known cancer outcome to train the MTR pipeline to assign a posterior probability of cancer risk to individual patches extracted from a mammogram. The MTR pipeline used in the present study was trained on data from three different independent populations. The first two were used in earlier texture studies [30, 31] and the third consisted of a case/control study similar to the current one, but using 2006 data and including 93 cases and 86 controls. The aggregate risk of a new mammogram is the average MTR posterior across extracted patches – typically 500 patches/scores per mammogram. The technical details can be found in [42]. An average of the CC and MLO projection was used to denote the automated MTR breast score. For the 4 women with only MLO images available, CC measures were estimated using linear regression.

In order to assign a single final score per woman for each method, the highest risk score was used if the two breasts differed. This approach is also normal procedure in the Copenhagen routine mammography screening programme, just as it is stipulated by ACR [14]. Fundamentally, the Tabár classification is not categorised according to a continuous risk scale. Based on risk evaluation available from the literature we ranked the Tabár classification as follows: *PII, PIII, PI, PV, PIV* where the low-risk patterns PI-PIII were ranked based on increasing density [29, 41, 43].

### Statistical analysis

#### Group characteristics for cases versus controls

Mean and 95 % CI were calculated for cases and controls separately regarding BI-RADS, MTR and age at screen, and group characteristics were compared using linear mixed model for analysis of matched pairs.

#### Association between methods

Median and inter-quartile range of MTR for each of the four BI-RADS and five Tabár categories as well as their combined subgroups were calculated. The pair-wise relations between methods were also demonstrated graphically using bar charts and box-and-whisker plots. The correlation between BI-RADS and Tabár was evaluated using Fisher’s exact test and Cramer’s V, and the correlation between MTR scores and the ordinal BI-RADS classification was evaluated using Spearman’s rho. Differences in MTR scores for each BI-RADS or Tabár category after stratification on case–control status were evaluated using linear mixed models for analysis of matched pairs (including age at screen as a co-variant).

#### Association with breast cancer

The ability of each individual method to separate cases from controls were evaluated using 1) logistic regression to calculate Odds Ratios (ORs) and 2) area under the receiver operating characteristic curves (AUCs). To calculate ORs similar to the two categorical classifications, the continuous MTR measure was categorised using cut-offs from the quartiles of control subjects. For all methods, each density/texture group was compared individually with the most fatty breast or lowest quartile (reference): D1 for BI-RADS, PII for Tabár, and the lowest quartile for MTR. We intended to base this study on information always available at screening—the woman’s age and her mammogram. Thus, only age at screening was adjusted for in the multivariate analysis, as information on body mass index (BMI) and other known risk factors for breast cancer are not collected routinely.

Moreover, we investigated the potential gain in prediction of breast cancer when using information from multiple methods in conjecture. To do so we used multiple logistic regression models, including main effects of various selections of predictors (age at screen, BI-RADS, Tabár and MTR). No interaction terms were found to be significant and these were therefore not included in the models. For each suggested model we computed AUCs based on its estimated linear predictor, and ORs for the model were reported by categorizing the linear predictor according to the quartiles of the controls. The statistical significance of differences between AUCs were assessed using the DeLong test [44].

IBM SPSS Statistics 20, Copyright © IBM Corporation 1989–2011, was used for statistical analysis and results were considered statistically significant with two-sided *P*-values < 0.05.

## Results

Table 1 shows the characteristics of cases and controls. Only a very small age difference (however significant) was seen between cases and controls (mean age of 57.9; 57.0–58.8 versus 58.2; 57.5–58.9, respectively) consistent with the age matched design on year of birth. From the 121 included cases 91 % were diagnosed with invasive breast cancer and the remaining with ductal carcinoma in situ (DCIS). Time from screening to diagnosis was 4 to 45 months with an average of 26 months. On average, cases demonstrated significantly higher BI-RADS density and automated texture scores than controls.

**Table 1:** Group characteristics for cases versus controls

	Cases^a^ (*n =* 121)		Controls (*n =* 259)		
	Mean	95 % Cl	Mean	95 % Cl	*P*-value^b^
Age	57.9	57.0–58.8	58.2	57.5–58.9	0.016
BI-RADS	2.44	2.24–2.64	1.98	1.86–2.10	<0.001
MTR	0.541	0.535–0.547	0.526	0.522–0.530	<0.001

Highest score of left or right breast for BI-RADS and MTR (MTR breast score: average of CC and MLO)

^a^DCIS 9.1 % and invasive cancer 90.9 %

^b^Statistics: Linear mixed model for matched pairs

**Fig. 3:**
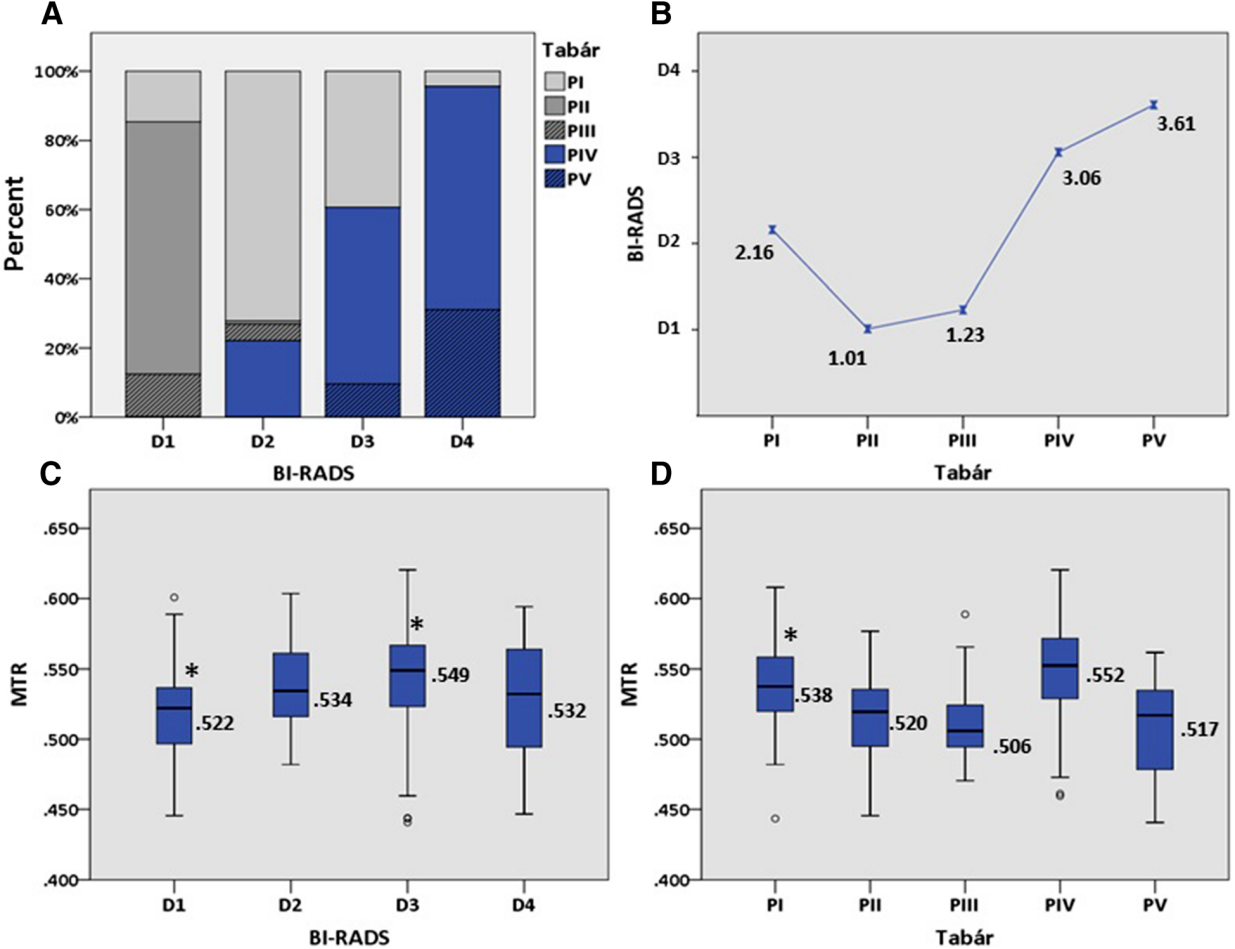
Pair-wise relation between three methods of assessing mammographic density or structural appearance (*n =* 380). **a** The proportional distribution of Tabár patterns within each BI-RADS category. **b** Mean BI-RADS score for each Tabár category. **c** Box-and-whisker plot showing the median (horizontal line), interquartile range (the box) and top + bottom 25 % of the scores except from outliers (whiskers) for the Mammographic Texture Resemblance scores for each BI-RADS category. **d** Box-and-whisker plot showing the MTR distribution for each Tabár category. *Significant difference between cases and controls

Table 2 summarizes the categorization of women into BI-RADS and Tabár patterns in a cross tabulation with corresponding median measures and inter-quartile ranges according to the automated texture scores. The pair-wise relations between the different methods are shown in Fig. 3. The BI-RADS and Tabár classifications were associated (*p* < 0.001) with Cramer’s V of 0.60 indicating a moderate association (Fig. 3a + b). Thus, women categorized into Tabár’s fatty PII and PIII were only seen in the two low-density BI-RADS categories (D1 + D2). Likewise, Tabár’s PIV and PV were mainly seen in the two high-density BI-RADS categories (D3 + D4). However, 23 women (6 %) with low density (D2) according to BI-RADS were classified with a high-risk nodular Tabár PIV. Tabár’s PI were distributed into all four BI-RADS categories but concentrated in the two middle categories – primarily D2. As demonstrated in Fig. 3c the automated texture scores increased with increasing BI-RADS density, however, with a drop in MTR scores as regards the extremely dense breasts (Spearman’s rho = 0.27; 0.17-0.37). A similar pattern was seen when the MTR scores were related to the five Tabár categories (Fig. 3d). The lowest texture scores were observed for the fatty PII and PIII breasts and increased for PI and even more for PIV, which demonstrated the highest MTR scores. A pronounced decrease in texture was seen for PV. When stratified into cases and controls, we saw a tendency for cases to reveal higher texture scores than controls in the three least dense BI-RADS categories (D1-D3) and the following Tabár categories: PI, PII and PIII (significant for category D1, D3 and PI).

**Table 2:** Distribution of BI-RADS and Tabár patterns with corresponding median measures of MTR in 380 women

		BI-RADS			
	Total:	D1	D2	D3	D4
	380 (0.32)	137 (0.22)	104 (0.30)	94 (0.39)	45 (0.51)
	*0.532 (0.508–0.554)*	*0.522 (0.496–0.537)*	*0.534 (0.515–0.561)*	*0.549 (0.523–0.567)*	*0.532 (0.494–0.564)*
Tabár					
I	134 (0.28)	20 (0.35)	75 (0.25)	37 (0.28)	2 (0.50)
	*0.538 (0.520–0.558)*	*0.538 (0.528–0.557)*	*0.535 (0.511–0.560)*	*0.540 (0.520–0.555)*	*0.528 (0.511–0.545)* ^*a*^
II	101 (0.18)	100 (0.17)	1 (1.00)	0	0
	*0.520 (0.493–0.536)*	*0.519 (0.493–0.535)*	*0.562 (−)*	*-*	*-*
III	22 (0.41)	17 (0.35)	5 (0.60)	0	0
	*0.506 (0.494–0.524)*	*0.500 (0.491–0.524)*	*0.513 (0.496–0.541)*	*-*	*-*
IV	100 (0.49)	0	23 (0.35)	48 (0.52)	29 (0.55)
	*0.552 (0.529–0.572)*	*-*	*0.535 (0.527–0.565)*	*0.556 (0.544–0.577)*	*0.547 (0.497–0.570)*
V	23 (0.30)	0	0	9 (0.11)	14 (0.43)
	*0.517 (0.478–0.535)*	*-*	*-*	*0.509 (0.452–0.537)*	*0.521 (0.479–0.537)*

The proportion of cases is displayed in brackets next to the number of women in each cell

The median and inter-quartile range is shown in *italics* for the *MTR* score

^a^ The two values in this cell are shown in brackets instead of the inter-quartile range

Table 3 demonstrates how all three methods were able to segregate women into different risk groups. We found that the risk of breast cancer in terms of ORs adjusted for age were significantly higher for women with BI-RADS D3 and D4 (OR 2.37; 1.32–4.25 and 3.93; 1.88–8.20), Tabár’s PIII and PIV (OR 3.23; 1.20–8.75 and 4.40; 2.31–8.38) and the upper quartile (Q4) of the MTR score (3.04; 1.63–5.67). To enable comparison between the different methods, independent of reference category, AUCs were also calculated for each method. Age adjusted AUCs for BI-RADS, Tabár and MTR were 0.63 (0.57–0.69), 0.65 (0.59–0.71) and 0.63 (0.57–0.69) (continuous), respectively.

**Table 3:** Association between mammographic density/structural appearance and breast cancer in 380 screening women^a^

	Cases/controls (*n*)	Case-ratio	OR (95 % Cl)^b^	*p*-value	AUC (95 % Cl)^b^
BI-RADS					0.63 (0.57–0.69)
D1	30/107	21.9	1 (reference)	-	
D2	31/73	29.8	1.53 (0.85–2.75)	NS	
D3	37/57	39.4	2.37 (1.32–4.25)	0.004	
D4	23/22	51.1	3.93 (1.88–8.20)	<0.001	
Tabár					0.65 (0.59–0.71)
PI	38/96	28.4	1.81 (0.96–3.42)	NS	
PII	18/83	17.8	1 (reference)	-	
PIII	9/13	40.9	3.23 (1.20–8.75)	0.021	
PIV	49/51	49.0	4.40 (2.31–8.38)	<0.001	
PV	7/16	30.4	1.97 (0.70–5.57)	NS	
Automated texture (MTR)^c^					0.63 (0.57–0.69) cont.
					*0.63 (0.56–0.69) cat.*
Q1	19/65	22.6	1 (reference)	-	
Q2	24/65	27.0	1.27 (0.63–2.54)	NS	
Q3	21/65	24.4	1.11 (0.54–2.25)	NS	
Q4	57/64	47.1	3.04 (1.63–5.67)	<0.001	
BI-RADS + MTR					0.66 (0.60–0.72)
Q1	11/64	14.7	1 (reference)	-	
Q2	23/65	26.1	2.06 (0.93–4.57)	NS	
Q3	33/66	33.3	2.91 (1.36–6.25)	0.006	
Q4	54/64	45.8	4.91 (2.35–10.24)	<0.001	
BI-RADS + Tabár					0.67 (0.61–0.72)
Q1	13/64	16.9	1 (reference)	-	
Q2	19/64	22.9	1.46 (0.67–3.21)	NS	
Q3	30/66	31.3	2.24 (1.07–4.67)	0.032	
Q4	59/65	47.6	4.47 (2.24–8.94)	<0.001	
Tabár + MTR					0.68 (0.62–0.73)
Q1	13/65	16.7	1 (reference)	-	
Q2	22/65	25.3	1.69 (0.79–3.64)	NS	
Q3	24/65	27.0	1.85 (0.87–3.94)	NS	
Q4	62/64	49.2	4.84 (2.43–9.66)	<0.001	
BI-RADS + MTR + Tabár					0.69 (0.63–0.74)
Q1	12/64	15.8	1 (reference)	-	
Q2	16/66	19.5	1.29 (0.57–2.95)	NS	
Q3	28/65	30.1	2.30 (1.08–4.91)	0.032	
Q4	65/64	50.4	5.42 (2.67–10.98)	<0.001	

^a^BI-RADS and Tabár are based on consensus scores between two readers. Regarding all three methods the maximum breast score has been used as the woman’s final score (Tabár ranked as follows: PII, PIII, PI, PV, PIV). Regarding MTR an average of CC and MLO was used as the breast score

^b^ORs and AUCs are adjusted for age. A significant difference in AUC was seen for BI-RADS + Tabár + MTR versus BI-RADS and BI-RADS + Tabár + MTR versus MTR

^c^Cut points for MTR scores are based on an equal number of controls in each group: Q1) 0–0.5047, Q2) 0.5047-0.5284, Q3) 0.5284-0.5469, Q4) 0.5469-1.00

The baseline AUC of 0.63 for BI-RADS density increased to 0.66–0.67 (non-significantly) when combining BI-RADS with either of the two other measures (Tabár or MTR). Combining all three measures increased AUC slightly more to 0.69 (0.63–0.74), which was significantly different from BI-RADS and texture alone. ORs based on the categorized new linear predictors from the combination models are also shown in Table 3.

## Discussion

Screening for breast cancer is entering an era of personalized screening. Hence, mammography screening is moving from the “one-size-fits-all” towards tailored screening strategies based on a woman’s risk profile (including density) [10, 12]. In Denmark—as in many other countries—population-based breast cancer screening is today based solely on the age of the woman. The only exception is intensified screening for the small subset of women belonging to families with moderately/highly increased lifetime risk (>30 %) or high-susceptibility genes as *BRCA1* and *BRCA2*. In a previous study we investigated inter-observer agreement regarding three subjective methods for density assessment [38]. In that study we addressed the current concerns about reproducibility if subjective methods are used to separate screening women. In the current study (based on the same case/control population) we focused on whether different methods may complement each other in risk assessment of screening women. Accordingly, we addressed whether it is relevant to distinguish between the (relative) *amount* of mammographic fibroglandular tissue (density)—BI-RADS scores—and the mammographic *structural appearance* (parenchymal pattern/texture)—Tabár and MTR scores—when determining the risk of future breast cancer. We found that all three methods were significantly associated with the risk of breast cancer. Furthermore, we demonstrated a significant improvement of the risk model when all three methods were combined into one aggregate measure of mammographic risk compared with density or texture alone. Even though, only a seemingly modest increase in discriminatory power was seen from an AUC of 0.63 for BI-RADS alone, to 0.66-0.67 when combining BI-RADS with either of the two other measures, and to 0.69 when combining all three measures, the AUCs must be regarded in the light of population-based screening. Even small improvements may have an impact at the population level, which was also demonstrated by the increasing gradient in breast cancer risk for the combination models seen in Table 3. Several studies have similarly found that adding new risk factors to already existing risk models only tends to show a modest increase in the discriminatory power [11, 45–48]. However, this remains of importance in outlining high-risk groups on a population basis [49]. Our results indicated that the three measures most likely captured different aspects of breast cancer risk, suggesting that a combined measure of density and structural appearance may well improve mammographic risk assessment in a future personalized population-based screening setting.

Overall, ORs were comparable with previous studies using identical density measures. The association between breast density and breast cancer risk as well as screening sensitivity has been well established in numerous previous studies [9, 50, 51]. In a prospective study, including more than 60,000 women followed for an average of 3.1 years, Vacek and Geller (2004) reported age-adjusted relative risks based on the BI-RADS density classification (D4 vs. D1) of 4.61 for premenopausal women and 3.88 for postmenopausal women [52]. Correspondingly, in a prospective cohort of 1 million women, Barlow and colleagues (2006) reported ORs of 3.93 and 3.15, respectively [11]. This is consistent with our OR of 3.93 for D4 versus D1 in predominantly postmenopausal women.

Few studies have investigated breast cancer risk applying the Tabár classification. Jakes et al. (2000) found unadjusted ORs of 2.30 (1.14–4.63) for PIV and 1.63 (0.72–3.68) for PIII using PI instead of the fattiest breast (PII) as a reference [43], which is well in accordance with our results giving ORs of 2.43 (1.41–4.18) for PIV and 1.78 (0.70–4.57) for PIII when PI is used for comparison. They demonstrated consistent ORs for the nodular PIV when individually adjusting for other risk factors. In addition, Jakes et al. did not observe any increased risk for PV (OR 0.78; 0.40–2.08), just as we did not find this pattern to be associated with increased risk of breast cancer (OR 1.09 (0.41–2.87) for PV versus PI).

Finally, risk segregation using the automated texture quantification technique was comparable with previous findings using earlier versions of the software [30, 31]. Based on a Dutch population, age-adjusted ORs for Q4 versus Q1 was 3.4 (2.1–5.8) (using cross-validation) and MTR scores were found to be independent of area percentage density [30]. This was supported by a subsequent study yielding an OR of 2.2 (1.4-3.6) for Q4 versus Q1 (when adjusted for BMI, age at menopause and postmenopausal hormone use). This study demonstrated that MTR generalizes as an independent risk factor (texture was estimated using training data from another cohort) [31]. The comparable ORs with previous findings are indicative of a general applicability of all three methods.

The underlying biological linkage between mammographic density (or density features) and breast cancer risk remains largely unresolved. Overall, a mammogram can be dominated by 1) fat 2) nodular/linear densities in varying amounts with potential biological (proliferative) activity and 3) homogeneous fibrous densities. In our study, the three methods largely agreed on the fatty breasts. Thus, BI-RADS D1 consisted mainly of fat involved PII and PIII breasts (Fig. 3a) and, in accordance; these predominantly structureless categories all revealed low texture scores (Fig. 3c and d). However, regarding mammograms with increasing density (mammograms with more structure, BI-RADS D2-D4) it was seen that they changed from being dominated by the “normal” Tabár PI pattern (in D2) to comprising the homogeneous dense PV pattern on behalf of PI (in D4). Moreover, the relative proportion of PIV patterns increased with increasing density (Fig. 3a). Thus, the more fibroglandular tissue on a mammogram the greater the risk of being categorized with a more aggressive looking PIV (or otherwise categorized as PV dominated by fibrosis which may or may not be associated with underlying proliferative activity). Taking the MTR scores into account it was illustrated how texture increases with increasing BI-RADS density but then decreases again for the extremely dense breasts (D4) (Fig. 3b). This can be due to D4 consisting of relatively more PV patterns with less structural features. The moderately dense breasts (D2 + D3) consist primarily of PI and PIV categories with the largest relative proportion of PIV in D3 breasts. The increase in texture scores from D2 to D3 and the fact that PIV reveals the highest texture scores suggests that MTR can distinguish breasts with a more aggressive pattern (PIV) from breasts with a less aggressive pattern (PI).

In general, we saw increasing ORs with increasing BI-RADS density (significant for D3 + D4) and correspondingly for Tabár PII- > PI- > PIV (significant for PIV). Similarly, MTR Q4 scores were significantly associated with increased risk. For all methods the fattiest (most structureless) breasts—which are also the easiest to read radiologically—were associated with lowest risk. The enlarged nodular and linear densities characteristic of Tabár’s PIV has been associated with a variety of benign changes of the breast parenchyma [41], and an inverse association with parity has been demonstrated [43, 53]. Interestingly, no significantly increased risk for Tabar PV was captured. This can be explained by the relatively few women categorized with this pattern (6 %), but might also be due to the structureless appearance. In addition, it could be attributed to misclassification into PV instead of PI. We also demonstrated increased ORs for Tabár’s PIII (supported by equivalent findings by Jakes et al., 2000). PIII is a fat involved breast, but is occupied by a retroareolar prominent duct pattern which—similar to PIV—has a more “aggressive” radiological appearance. However, MTR scores were not increased in regards to this specific pattern, presumably because this technique is based on average measures from numerous patches throughout the entire breast. In general, cases showed higher MTR scores than controls regarding all low-density patterns (BIRADS D1-D3 and Tabár PI-PIII) and 28 cases were identified in low density breasts. This indicates that the MTR technique captures a mammographic detectable risk that is different from risk due to density alone (Fig. 1). Thus, different features of breast morphology (amount, composition and organization of breast tissue) appear to be retrieved by the three various methods capturing different elements of risk. We didn’t observe any difference in cancers identified by the three methods according to DCIS/invasive-status.

In tailored screening, masking plays a significant role. Accordingly, women with high density might benefit from supplementary imaging with e.g. ultrasound, tomosynthesis, MRI or altered screening intervals. The fifth edition of BI-RADS no longer indicates quartiles of percentage dense tissue [14]. This has been done to put an emphasis on the masking potential of different density patterns as opposed to percentage breast density being an indicator for breast cancer risk. Tabár has also emphasised the masking potential for pattern IV and V rather than a biological risk [41]. However, data from the Swedish Kopparberg randomized controlled trial showed a RR of 1.57 (1.23–2.01) for dense (PIV and PV) versus non-dense (PI, PII and PIII) mammograms after 25 years of follow-up, and a recent study found the association between mammographic density and breast cancer risk to persist up to 10 years after the baseline mammogram [8, 54]. Thus, the increased risk from baseline breast density patterns seems to remain after long-time follow-up indicative of an inherent risk which cannot be explained by the masking effect. In our study the BI-RADS D4 category showed the greatest masking potential of all groups. Thus, 39 % of the cancers in this category were diagnosed before the woman’s next regular screen (<2 years from baseline screen); an even higher proportion were seen for the combined D4/PIV subgroup (44 % - data not shown). Correspondingly, we saw that effect sizes increased quite notably, especially when using the BI-RADS and Tabár classification, when only looking at cancers diagnosed < 2 years from the baseline screening (Additional file 1). This suggests that certain BI-RADS and Tabár patterns, in particular, are strongly indicative of the potential of masking. However, all three methods were still able to stratify women into the risk of future breast cancer (cancers diagnosed ≥2 years from baseline; Additional file 1).

### Limitations

Our study has some limitations. First of all, the sample size of included women is rather small leading to wide confidence intervals and restricting stratification into subgroups. Next, two subjective methods were investigated introducing uncertainty about reproducibility. However, we used consensus scores from two independent readers which had demonstrated substantial inter-observer agreement for both methods [38]. Both readers had no previous experience using the Tabár classification and only one of the readers had experience from clinical mammography (not screening) regarding the BI-RADS classification. The lack of experience only adds to robustness of the classifications and the ORs found in this study. We also have a relatively short follow-up period of 3–4 years. A small study by van Gils et al. (1998) found the effect of masking to be small but to peak 3–4 years after the initial screening [55]. In addition, we did not control for any other risk factors or confounders (except from age) in this retrospective study which might have influenced our risk estimates. In particular, BMI has been reported an important confounder especially among postmenopausal women, and adjusting for BMI would expectably have led to some increase in OR estimates [51, 52, 56]. However, the lack of further adjustments is equal for all the methods being compared. Besides, we intended to base our study exclusively on data available at screening. From a clinical point of view, our results are more directly applicable in present screening programs where the mammogram in addition to the woman’s age is the only available information to the radiologist.

## Conclusions

This study confirms the increased risk of breast cancer associated with high mammographic density (BI-RADS D3 and D4), Tabár’s PIV and high measurements of mammographic texture. Furthermore, it provides more evidence that mammographic structural features and density can be considered independent biomarkers for breast cancer risk. Both Tabár and MTR identify women at increased risk of breast cancer who have low density, and our study suggests that breast cancer risk may be attributable to different mammographic features captured by each of the three methods. However, it might not be feasible to introduce more classifications for radiologists to adapt and apply in a busy and comprehensive screening environment. A combined—and optimally automated—measure of density and texture could form the basis of a future prospective validation study, which evaluates the impact of risk based stratification on breast cancer diagnosis, false positive rate, and breast cancer mortality. This could be moving closer to an applicable mammographic risk marker in population-based screening, in respect to a potential future individualized screening set-up.

## Abbreviations

ACR, the American College of Radiology; AUC, area under the ROC curve; BI-RADS, Breast Imaging Reporting and Data System; BMI, body mass index; CC, craniocaudal; CI, confidence interval; DCIS, ductal carcinoma in situ; HRT, hormone replacement treatment; ICC, Intraclass Correlation Coefficient; MLO, mediolateral oblique; MTR, mammographic texture resemblance; OR, odds ratio; PMD, Percentage Mammographic Density; ROC curve, receiver operating characteristic curve.

## Additional file

Additional file 1: ORs for cancers diagnosed before or after 2 years from baseline screening, respectively. (DOC 60 kb)
