# Supplementary material for: Mammographic density and structural features can individually and jointly contribute to breast cancer risk assessment in mammography screening: a case–control study
Source: BMC Cancer. 2016 Jul 7;16:414. doi: 10.1186/s12885-016-2450-7 (PMC4936245; doi:10.1186/s12885-016-2450-7)
Supplement: Additional file 1: — ORs for cancers diagnosed before or after 2 years from baseline screening, respectively. (DOC 60 kb) [file 12885_2016_2450_MOESM1_ESM.doc]

**Additional file 1** ORs for cancers diagnosed before or after 2 years from baseline screening, respectively.

|  | **Cases/controls**  **(case-ratio)** | **OR***  **(95% CI)** | **p-value** | **AUC***  **(95% CI)** |
| --- | --- | --- | --- | --- |
| **Cancers diagnosed before next regular screen (<2 years from baseline screen) n=99** | | | | |
| **BI-RADS** |  |  |  | 0.70 (0.59-0.81) |
| **D1** | 5/26 (0.16) | 1.00 | - |  |
| **D2** | 8/16 (0.33) | 2.71 (0.75-9.81) | NS |  |
| **D3** | 12/17 (0.41) | 3.91 (1.15-13.30) | 0.029 |  |
| **D4** | 9/6 (0.60) | 9.51 (2.15-42.14) | 0.003 |  |
| **Tabár** |  |  |  | 0.73 (0.62-0.83) |
| **PI** | 13/19 (0.41) | 7.73 (1.51-39.54) | 0.014 |  |
| **PII** | 2/21 (0.09) | 1.00 | - |  |
| **PIII** | 1/5 (0.17) | 1.96 (0.15-26.42) | NS |  |
| **PIV** | 16/14 (0.53) | 12.91 (2.51-66.51) | 0.002 |  |
| **PV** | 2/6 (0.25) | 3.97 (0.44-35.62) | NS |  |
| **Texture (MTR)**** |  |  |  | 0.67 (0.55-0.78) cont *0.66 (0.54-0.78) cat.* |
| **Q1** | 5/22 (0.19) | 1.00 | - |  |
| **Q2** | 7/14 (0.33) | 2.15 (0.57-8.17) | NS |  |
| **Q3** | 5/12 (0.29) | 1.85 (0.44-7.72) | NS |  |
| **Q4** | 17/17 (0.50) | 4.54 (1.38-15.00) | 0.013 |  |
| ***Cancers diagnosed after next regular screen (≥2 years from baseline screen, n=259)*** | | | | |
| **BI-RADS** |  |  |  | 0.61 (0.54-0.68) |
| **D1** | 25/71 (0.26) | 1.00 | - |  |
| **D2** | 23/52 (0.31) | 1.26 (0.65-2.47) | NS |  |
| **D3** | 25/36 (0.41) | 2.04 (1.02-4.08) | 0.044 |  |
| **D4** | 14/13 (0.52) | 3.28 (1.31-8.17) | 0.011 |  |
| **Tabár** |  |  |  | 0.64 (0.57-0.72) |
| **PI** | 25/69 (0.27) | 1.22 (0.59-2.51) | NS |  |
| **PII** | 16/54 (0.23) | 1.00 | - |  |
| **PIII** | 8/8 (0.50) | 3.42 (1.10-10.64) | 0.034 |  |
| **PIV** | 33/31 (0.52) | 3.58 (1.70-7.53) | 0.001 |  |
| **PV** | 5/10 (0.33) | 1.66 (0.49-5.64) | NS |  |
| **Texture (MTR)**** |  |  |  | 0.62 (0.55-0.69) cont *0.62 (0.55-0.69) cat.* |
| **Q1** | 14/41 (0.26) | 1.00 | - |  |
| **Q2** | 17/43 (0.28) | 1.16 (0.51-2.65) | NS |  |
| **Q3** | 16/46 (0.26) | 1.02 (0.45-2.35) | NS |  |
| **Q4** | 40/42 (0.49) | 2.80 (1.33-5.89) | 0.007 |  |

*adjusted for age

**based on cut-offs from the complete dataset on controls
